# Spatio-Temporal Patterns of Ticks and Molecular Survey of *Anaplasma marginale*, with Notes on Their Phylogeny

**DOI:** 10.3390/microorganisms10081663

**Published:** 2022-08-17

**Authors:** Shumaila Alam, Mehran Khan, Abdulaziz Alouffi, Mashal M. Almutairi, Shafi Ullah, Muhammad Numan, Nabila Islam, Zaibullah Khan, Ome Aiman, Sher Zaman Safi, Tetsuya Tanaka, Abid Ali

**Affiliations:** 1 Department of Zoology, Abdul Wali Khan University Mardan, Mardan 23200, Pakistan; 2 King Abdulaziz City for Science and Technology, Riyadh 12354, Saudi Arabia; 3 Department of Pharmacology and Toxicology, College of Pharmacy, King Saud University, Riyadh 11451, Saudi Arabia; 4 Department of Chemistry, Abdul Wali Khan University Mardan, Mardan 23200, Pakistan; 5 Faculty of Medicine, Bioscience and Nursing, MAHSA University, Jenjarom 42610, Selangor, Malaysia; 6 Laboratory of Infectious Diseases, Joint Faculty of Veterinary Medicine, Kagoshima University, 1-21-24 Korimoto, Kagoshima 890-0065, Japan

**Keywords:** hard ticks, *Anaplasma marginale*, surveillance, phylogeny, Pakistan

## Abstract

Hard ticks (Ixodida: Ixodidae) are medically important ectoparasites that feed on all classes of terrestrial vertebrates. Recently, we molecularly characterized hard ticks and associated *Anaplasma* spp. in the northern and central regions of Khyber Pakhtunkhwa (KP), Pakistan; however, this knowledge was missing in the southern regions. This study aimed to investigate tick prevalence, host range, genetic diversity, and molecular survey of *Anaplasma* spp. in a wide range of tick species in two distinct physiographic regions of southern KP. A total of 1873 hard ticks were randomly collected from 443/837 hosts (cattle, Asian water buffaloes, horses, goats, sheep, dogs, and camels) in Lakki Marwat, Bannu, and Orakzai districts of KP. Overall, 12 tick species were morphologically identified, among which *Hyalomma dromedarii* was the most prevalent species (390/1873, 20.9%), followed by *Hy. anatolicum* (294, 15.7%), *Rhipicephalus microplus* (262, 14%), *Hy. scupense* (207, 11.1%), *R. sanguineus* (136, 7.3%), *R. turanicus* (121, 6.5%), *Haemaphysalis cornupunctata* (107, 5.7%), *R. haemaphysaloides* (110, 5.9%), *Ha. montgomeryi* (87, 4.6%), *Hy. isaaci* (58, 3.1%), *Ha. bispinosa* (54, 2.9%), and *Ha. sulcata* (47, 2.5%). The extracted DNA from a subset of each tick species was subjected to PCR to amplify *cox1* or *16S rRNA* sequences of ticks and *16S rRNA* sequences of *Anaplasma* spp. The tick *cox1* sequences showed 99–100% identities with the sequences of the same species, whereas *16S rRNA* sequences of *R. turanicus*, *Ha. montgomeryi* and *Ha. sulcata* showed 97–100% identities with the corresponding species. The *16S rRNA* sequence of *Ha. cornupunctata* showed 92% identity with the species from the same subgenus, such as *Ha. punctata*. The *16S rRNA* sequence of *Anaplasma* spp. showed 100% identity with *Anaplasma marginale*. Moreover, 54 ticks were found positive for *A. marginale* with a total infection rate of 17.2%. The highest infection rate was recorded in *Hy. dromedarii* (31.1%) and the lowest in each *R. haemaphysaloides* and *R. sanguineus* (20%). All the *cox1* or *16S rRNA* sequences in phylogenetic trees clustered with the same species, except *Ha. cornupunctata*, which clustered with the *Ha*. (*Aboimisalis*) *punctata*. In this study, *Ha. cornupunctata* was reported for the first time at the molecular level. The genetic characterization of ixodid ticks and molecular detection of associated *A. marginale* will assist in the epidemiological surveillance of these parasites in the region.

## 1. Introduction

Ticks are obligatory blood feeders that infest terrestrial and semi-aquatic vertebrates in tropical and subtropical regions [1,2,3]. Ticks damage their hosts through several mechanisms, including the transmission of different disease-causing agents such as bacteria (*Anaplasma*, *Borrelia*, *Ehrlichia,* and *Rickettsia*), viruses (Bunyaviridae, Iridoviridae, and Reoviridae), and protozoans (*Babesia* and *Theileria*) [4].

According to the Global Climate Risk Index 2021, Pakistan is the eighth country prone to climate change [5]. The distribution patterns of ticks will be highly affected within such a region [6] and may favor the transmission of tick-borne pathogens (TBPs) [2,7]. However, there are few reports about tick distribution in different zones of Pakistan [2,3,7,8,9,10,11,12]. These studies have described the abundance of *Rhipicephalus*, *Hyalomma*, and *Haemaphysalis* ticks infesting animals and humans in Pakistan. Additionally, *Amblyomma* (*Am. gervaisi*, *Am. javanense*), *Ixodes* (*I. hyatti* and *I. redikorzevi*), *Ornithodoros* (*Pavlovskyella*) spp. (an undetermined species), and *Nosomma* (*N. monstrosum*) are rarely reported tick genera in the country [10,12,13,14,15,16]. The commonly reported species of the genus *Rhipicephalus* are: *R. haemaphysaloides*, *R. microplus*, *R. annulatus*, *R. turanicus,* and *R. sanguineus*. In contrast, the *Hyalomma* genus is comprised of *Hy. anatolicum*, *Hy. isaaci*, *Hy. scupense*, and *Hy. dromedarii* and species of genus *Haemaphysalis* are *Ha. cornupunctata*, *Ha. montgomeryi*, *Ha. kashmirensis*, *Ha. bispinosa*, and *Ha. sulcata*, with varying prevalence in different ecological regions of the country [2,3]. The most important TBPs causing animal health issues in Pakistan include species of *Anaplasma, Babesia*, and *Theileria* [2,17,18].

The pathogenic agents of anaplasmosis are highly prevalent worldwide, particularly in tropical and subtropical regions [19]. These pathogens have a wide genetic range and adversely affect the livestock industry [20,21]. Knowing that this field of research has attracted attention, there are still very few available studies restricted to a few areas in Pakistan about the molecular data of ticks and *Anaplasma* spp. [2,8,13,18,22,23,24]. Our previous study has demonstrated the molecular assessment of hard ticks and associated *A. marginale* collected from livestock hosts in the northern and central regions of Khyber Pakhtunkhwa (KP), Pakistan [24]. Still, similar studies are missing from the southern regions. This study aimed to investigate tick prevalence, genetic diversity, and molecular survey of associated *Anaplasma* spp. in a wide range of tick species in two distinct physiographic regions of southern KP.

## 2. Materials and Methods

### 2.1. Ethical Approval

Before this study, ethical approval was taken from the Advanced Studies and Research Board (Dir/A&R/AWKUM/2020/4871) of the Faculty of Chemical and Life Sciences, Abdul Wali Khan University Mardan, KP, Pakistan. Furthermore, written and/or oral consents were obtained from the animals’ owners for tick collection.

### 2.2. Study Area

**Figure 1:**
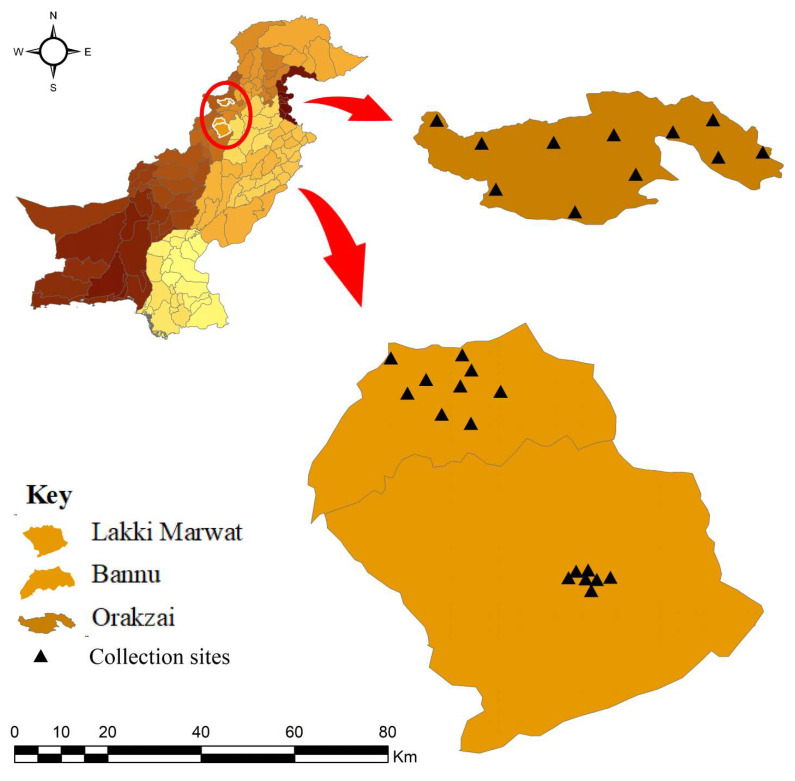
Map showing tick collection sites in southern Khyber Pakhtunkhwa, Pakistan.

The current study investigated three districts of southern KP, including Lakki Marwat (32.6135° N, 70.9012° E), Bannu (32.9910° N, 70.6455° E), and Orakzai (33.6671° N, 70.9547° E). These districts belong to two distinct physiographic regions, one with a “hot semi-arid climate” (Bannu and Lakki Marwat) and the other with a “humid subtropical climate” (Orakzai). Based on the ecological zones, the former is mainly a “desert plain,” and the latter is mainly a semi-arid piedmont. The geographic coordinates of each collection site were obtained using Global Positioning System (GPS) and loaded into a Microsoft Excel sheet to design a map using ArcGIS 10.3.1.3 (ESRI, Redlands, CA, USA) (Figure 1).

### 2.3. Tick Collection and Preservation

**Figure 2:**
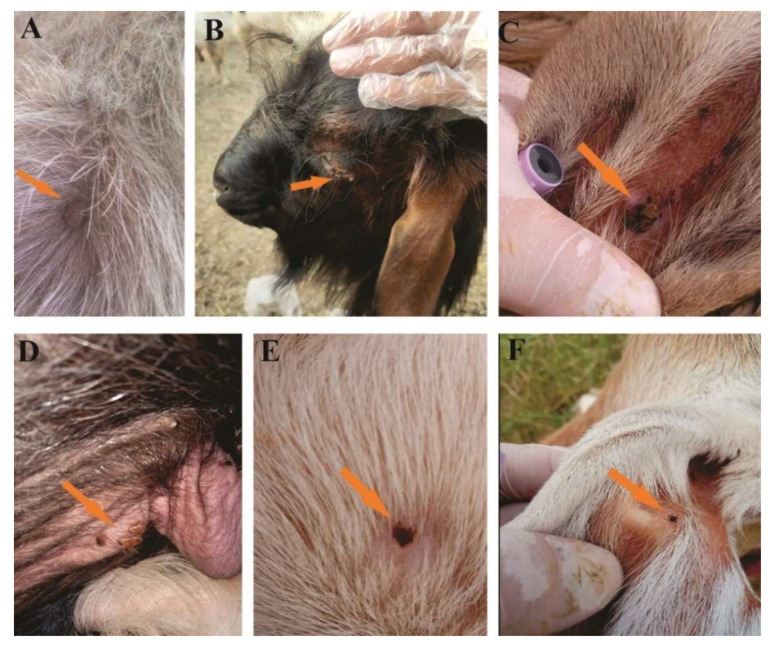
Tick infestation on different hosts: *Hy. dromedarii* from camels (**A**). *Ha. bispinosa* from goats (**B**). *R. turanicus* from sheep (**C**). *Hy. anatolicum* from male Asian water buffaloes (**D**). *R. sanguineus* from dogs (**E**). *R. turanicus* from goats (**F**).

Tick collection was carried out from March 2019 to February 2020 with a regular visit to the study area once a month. Ticks were randomly collected using forceps from different vertebrate hosts, including cattle, Asian water buffaloes, horses, goats, sheep, dogs, and camels (Figure 2). Tick specimens were rinsed with distilled water followed by 70% ethanol and were stored in 100% ethanol in properly labeled tubes for onward molecular experiments. During tick collection, the relevant information regarding collection date, host type, and place of collection of the ticks were noted.

### 2.4. Morphological Identification of Ticks

Ticks were morphologically identified using stereo zoom microscope (SZ61, Olympus Corporation, Tokyo, Japan) and standard morphological keys [6,25,26,27,28,29,30,31,32,33].

### 2.5. DNA Extraction and PCR

Before the genomic DNA extraction, ticks were washed with distilled water and dried on sterile filter paper. The ticks were crushed with sterilized pestles in 1.5 mL sterile Eppendorf tubes. Genomic DNA was extracted individually from each tick using the phenol–chloroform method according to the standard protocol. The DNA pellet was hydrated by adding 30 µL of nuclease-free water [34]. The quality and quantity of genomic DNA were determined through Nano-Q (Optizen, Daejeon, Korea).

By using reference primers and PCR conditions (Table 1), the extracted DNA was subjected to amplifying partial fragments of ticks *cox1* and *16S rRNA* genes and screened for *16S rRNA* of *Anaplasma* spp. in Table 2 through a PCR. Each PCR reaction was prepared in a 20 μL reaction mixture and contained: 12 μL of Dream*Taq* MasterMix (Thermo Fisher Scientific, Inc., Waltham, MA, USA), 1 μL of each forward and reverse primers (10 μM), 2 μL (50 ng/μL) genomic DNA template and 4 μL PCR water (nuclease-free). The DNA of *R. microplus* and *Rickettsia massiliae* were used as positive controls for ticks and *Anaplasma* spp., respectively, while PCR water (nuclease-free) was used as a negative control. The amplified DNA was run on a 1.5% agarose gel, dyed with 2 µL ethidium bromide, and observed by a Gel documentation system (BioDoc-It™ Imaging Systems UVP, LLC, Upland, CA, USA).

### 2.6. DNA Sequencing and Phylogenetic Analysis

Purification of PCR products was performed using GeneClean II Kit (Qbiogene, Illkirch, France) following the manufacturer’s protocol. A total of 64 (*cox1* 40 and *16S rRNA* 24) amplified PCR products for ticks and 18 (3 from each *Anaplasma* positive tick species amplicons) for *16S rRNA Anaplasma* spp. were submitted for bidirectional DNA sequencing (Macrogen, Inc., Seoul, South Korea). The sequences were cropped to remove the primers and poor reading regions through SeqMan V. 5 (DNASTAR). The obtained purified sequences were subjected to the Basic Local Alignment Search Tool (BLAST) [38] at National Center for Biotechnology Information (NCBI), and the homologous sequences were downloaded. These sequences were aligned with obtained sequences along with an outgroup in BioEdit Sequence Alignment Editor V. 7.0.5 (Raleigh, NC, USA) [39]. The phylogenetic trees were constructed by using the Maximum-Likelihood model (1000 bootstrap replicons) in Molecular Evolutionary Genetics Analysis (MEGA-X) [40].

## 3. Results

### 3.1. Morphologically Identified Ticks

The morphological identification confirmed 12 tick species belonging to the three genera of hard ticks. The genus *Hyalomma* included *Hy. dromedarii*, *Hy. anatolicum*, *Hy. scupense* and *Hy. isaaci*, the genus *Rhipicephalus* contained *R. microplus*, *R. sanguineus*, *R. haemaphysaloides*, and *R. turanicus*, while the genus *Haemaphysalis* included *Ha. montgomeryi*, *Ha. bispinosa*, *Ha. sulcata* and *Ha. cornupunctata*.

### 3.2. Prevalence of Ticks

A total of 1873 ticks were randomly collected from 443/837 infested hosts (cattle, Asian water buffaloes, horses, goats, sheep, dogs, and camels) comprising *Hyalomma* (949/1873, 50.6%), *Rhipicephalus* (629/1873, 33.6%), and *Haemaphysalis* (295/1873, 15.8%). Overall, *Hy*. *dromedarii* was the most prevalent species (390/1873, 20.8%), followed by *Hy. anatolicum* (294, 15.7%), *R. microplus* (262, 14%), *Hy. scupense* (207, 11%), *R. sanguineus* (136, 7.3%), *R. turanicus* (121, 6.5%), *Ha. cornupunctata* (107, 5.7%), *R. haemaphysaloides* (110, 5.9%), *Ha. montgomeryi* (87, 4.6%), *Hy. isaaci* (58, 3.1%), *Ha. bispinosa* (54, 2.9%), and *Ha. sulcata* (47, 2.5%). Detailed data about each tick species’ number and percentage of life stages are shown (Table 2).

### 3.3. Spatial Pattern of Ticks

**Figure 3:**
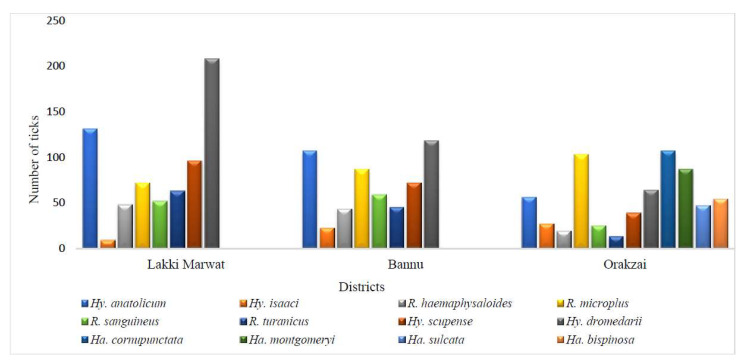
Spatial patterns of the collected ixodid ticks in the study regions.

The highest number of ticks were recorded from Lakki Marwat (679/1873, 36.3%), followed by Orakzai (641/1873, 34.2%), and Bannu (553/1873, 29.5%). Herein, eight tick species were reported representing two genera from Lakki Marwat in which *Hy. dromedarii* (208/679, 30.6%) was the most abundant, followed by *Hy. anatolicum* (131/679, 19.3%), and *Hy. scupense* (96/679, 14.1%). Eight tick species comprising two tick genera were recorded from the Bannu district in which *Hy. dromedarii* (118/553, 21.3%) was the most abundant species, followed by *Hy. anatolicum* (107/553, 19.3%), and *R. microplus* (87/553, 15.7%). In contrast, *Ha. cornupunctata* (107/641, 16.6%) was the most dominant species in the Orakzai district, followed by *R. microplus* (103/641, 16%) and *Ha. montgomeryi* (87/641, 13.5%). *Haemaphysalis* species were only found in the Orakzai district, while we could not collect these species in the other two districts. The details of each tick species reported from the study area are provided (Figure 3).

### 3.4. Seasonal Abundance of Ticks

**Figure 4:**
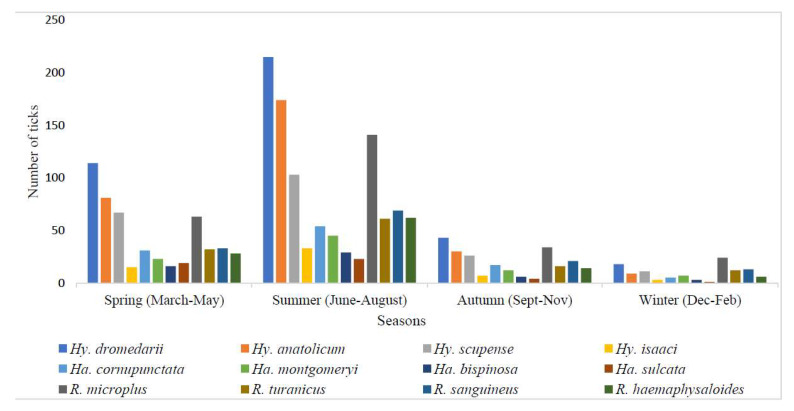
Seasonal abundance of the collected ixodid ticks in the study regions.

Tick abundance was highly fluctuated by seasonal variations. The highest number of ticks were reported in summer (June–August) (1009/1873, 53.9%), followed by spring (March–May) (522/1873, 27.9%), autumn (September–November) (230/1873, 12.3%), and winter (Dec–Feb) (112/1873, 5.9%) (Figure 4). Details about the seasonal abundance of each tick species in all four seasons are presented in the graph (Figure 4).

### 3.5. Detection of Anaplasma spp. in Ticks

*Anaplasma* spp. was detected in 54 out of 314 selected ticks with a total infection rate of 17.2% (54/314). Out of 12 examined tick species, *Anaplasma* spp. were detected in six species, such as *Hy. dromedarii*, *Hy. anatolicum*, *Hy. scupense*, *R. sanguineus*, *R. microplus* and *R. haemaphysaloides*. The highest infection rate was recorded in *Hy. dromedarii* 31.1% (14/45), followed by *R. microplus* 30% (12/40), *Hy. scupense* 27.3% (9/33), *Hy. anatolicum* 23.8% (10/42), and in each *R. haemaphysaloides* and *R. sanguineus* 20% (6/30), with no amplification of *Anaplasma* DNA in the selected *Haemaphysalis* species. The detailed information regarding the infection rate of the selected species is shown in Table 2.

### 3.6. Sequencing Analysis

From the extracted tick DNA of 12 tick species, the partial fragments of *cox1* were amplified for eight tick species, whereas *16S rRNA* was amplified for four tick species. Clean *cox1* sequences were obtained from eight tick species: *Hy. dromedarii* (743 bp), *Hy. anatolicum* (791 bp), *Hy. scupense* (775 bp), *Hy. isaaci* (771 bp), *Ha. bispinosa* (728 bp), *R. microplus* (800 bp), *R. sanguineus* (612 bp), and *R. haemaphysaloides* (797 bp), while *16S rRNA* sequences were obtained from four tick species: *R. turanicus* (398 bp), *Ha. cornupunctata* (394 bp), *Ha. montgomeryi* (265 bp) and *Ha. sulcata* (396 bp). The identical sequences were considered as a single consensus sequence. The BLAST results of the obtained *cox1* sequences of *Hy. dromedarii*, *Hy. anatolicum*, *Hy. scupense*, *Hy. isaaci*, *Ha. bispinosa*, *R. microplus*, *R. sanguineus*, and *R. haemaphysaloides* showed maximum identities of 99–100%, with the same species reported from Egypt, India, France, Pakistan, Bangladesh, and Iran. In the case of *16S rRNA*, the BLAST results of *R. turanicus*, *Ha. montgomeryi* and *Ha. sulcata* showed the highest identities of 99.75%, 96.99%, and 98.75%, respectively, with the same species reported from Afghanistan, China, and Pakistan, while the *16S rRNA* sequence of *Ha. cornupunctata* showed the maximum identity of 92% with the *Ha. punctata* reported from China, Turkey, Italy, Spain, and Portugal. The *16S rRNA* sequences (931 bp) of *Anaplasma* spp. were subjected to BLAST and showed 100% identity with the *A. marginale*.

### 3.7. Phylogenetic Analysis

The phylogenetic tree for the *cox1* sequences of *Hy. dromedarii*, *Hy. anatolicum*, *Hy. scupense*, *Hy. isaaci*, *Ha. bispinosa*, *R. microplus*, *R. sanguineus*, and *R. haemaphysaloides* were constructed combinedly with 49 sequences downloaded from NCBI based on the maximum identity. In the phylogenetic tree, the obtained *cox1* sequences were clustered to the corresponding species reported from different countries, such as *Hy. dromedarii* from Egypt and Tunisia, *Hy. anatolicum* from India, Egypt, and China, *Hy. scupense* from France, Spain, China, and Turkey, *Hy. isaaci* from Pakistan, *Ha. bispinosa* from India and Bangladesh, *R. microplus* from Pakistan, India, and China, *R. sanguineus* from Iran and India, and *R. haemaphysaloides* from Pakistan, India, and China. In the case of *16S rRNA*, the phylogenetic tree of *R. turanicus*, *Ha. cornupunctata*, *Ha. montgomeryi* and *Ha. sulcata* was constructed with 27 sequences downloaded from NCBI based on the maximum identity. In the phylogenetic tree, *16S rRNA* sequences of *R. turanicus*, *Ha. montgomeryi* and *Ha. sulcata* clustered with the same species reported from Afghanistan, Pakistan, and China, while *Ha. cornupunctata* clustered with the species of the same subgenus *Ha.* (*Aboimisalis*) *punctata* reported from China, Turkey, Italy, Spain, and Portugal.

**Figure 5:**
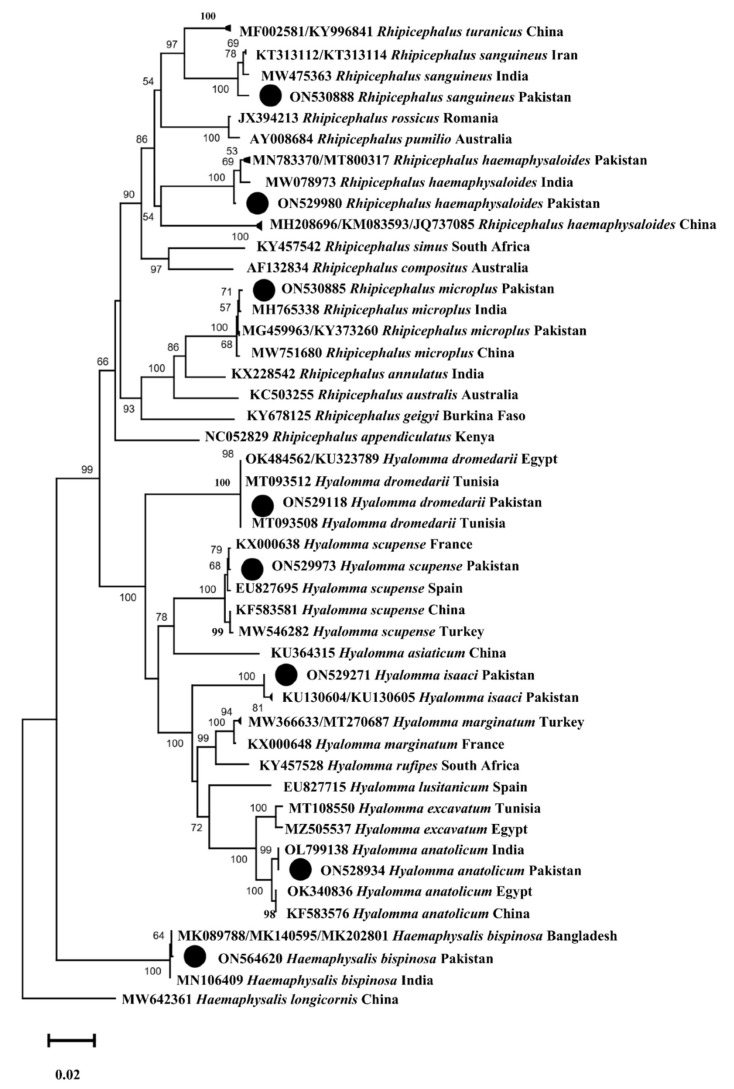
Maximum likelihood phylogenetic tree based on *cox1* sequences of *Hy. dromedarii*, *Hy. anatolicum*, *Hy. scupense*, *Hy. isaaci*, *Ha. bispinosa*, *R. microplus*, *R. sanguineus* and *R. haemaphysaloides*. *Haemaphysalis longicornis* was used as an outgroup, using supporting values (1000 replicons) at each node. The scale bar indicates the number of substitutions per site. The obtained sequences were represented with black circles.

**Figure 6:**
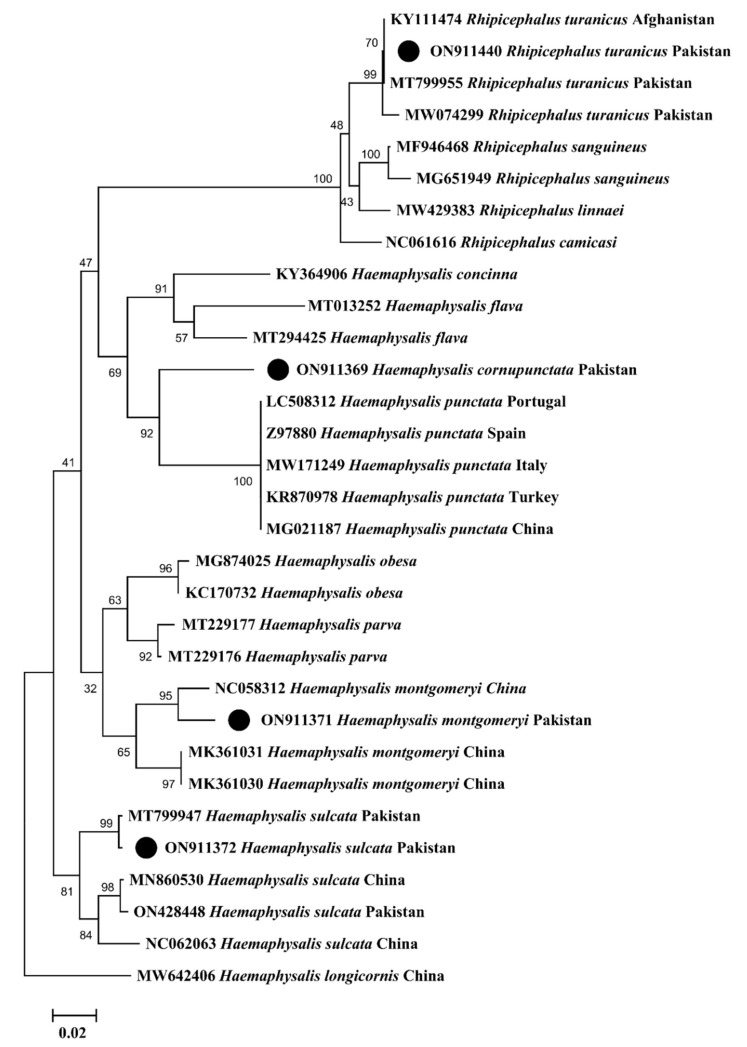
Maximum likelihood phylogenetic tree based on *16S rRNA* sequences of *R. turanicus*, *Ha. cornupunctata*, *Ha. montgomeryi* and *Ha. sulcata*. *Haemaphysalis longicornis* was used as an outgroup, using supporting values (1000 replicons) at each node. The scale bar indicates the number of substitutions per site. The obtained sequences were represented with black circles.

All the obtained *cox1* sequences were uploaded to the GenBank under accession numbers: ON529118 (*Hy. dromedarii*), ON528934 (*Hy. anatolicum*), ON529973 (*Hy. scupense*), ON529271 (*Hy. isaaci*), ON564620 (*Ha. bispinosa*), ON530885 (*R. microplus*), ON530888 (*R. sanguineus*), and ON529980 (*R. haemaphysaloides*). The obtained *16S rRNA* sequences were uploaded under accession numbers: ON911440 (*R. turanicus*), ON911369 (*Ha. cornupunctata*), ON911371 (*Ha. montgomeryi*), and ON911372 (*Ha. sulcata*). The phylogenetic trees of the obtained *cox1* and *16S rRNA* sequences are shown in Figure 5 and Figure 6, respectively.

**Figure 7:**
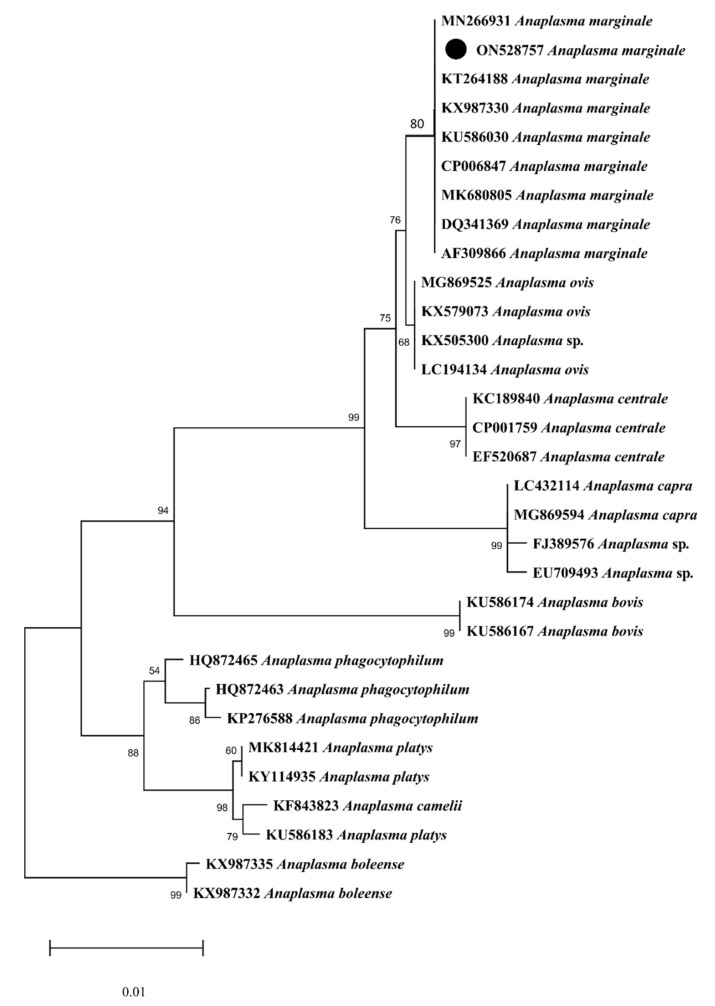
Maximum likelihood phylogenetic tree based on the partial *16S rRNA* sequence of *A. marginale*. The *Anaplasma boleense* was used as an outgroup, using supporting values (1000 replicons) at each node. The scale bar indicates the number of substitutions per site. The obtained sequence was represented with a black circle.

A total of 29 sequences of *16S rRNA* for *A. marginale* were downloaded from GenBank in FASTA format based on maximum identity with query sequences. In the phylogenetic tree, the obtained partial *16S rRNA* sequence of *A. marginale* clustered with the same sequences reported from Kenya, Thailand, Australia, Pakistan, and China (Figure 7). The obtained partial *16S rRNA* sequence of *A. marginale* was uploaded to the GenBank (ON528757).

## 4. Discussion

Pakistan has an agrarian economy where agriculture contributes approximately 21% to gross domestic product (GDP) and 45% to the labor force [41]. Ticks pose severe threats to the livestock and economy of the country. Knowledge regarding molecular surveillance of ticks and *A. marginale* and their host range in different physiographic is essential for implementing adequate measures against these parasites in Pakistan. The present study was executed in two distinct physiographic regions in southern KP, Pakistan. The targeted areas were selected because ticks and tick-borne diseases are common in these regions but mainly remained unexplored, and to compare tick diversity in two regions that are geographically close but physiographically and climatically different. Herein, 12 tick species were morphologically and molecularly identified. Four tick species, including *Ha. bispinosa*, *Ha. cornupunctata*, *Hy. dromedarii* and *Hy. isaaci* were genetically characterized for the first time from Pakistan. Furthermore, the molecular survey was conducted to screen a subset of the collected 12 species for *A. marginale*, in which this pathogen was detected in six species. Among these species, *A. marginale* was detected for the first time in *Hy. dromedarii*, *Hy. scupense*, *R. sanguineus* and *R. haemaphysaloides* from Pakistan.

Environmental and climatic factors influence the distribution and prevalence of ticks within a specific region [42]. Previous studies considered *Hyalomma* spp. as successful ticks in harsh desert regions [43,44]. Similarly, as a larger proportion of the current study area was a desert plain, the genus *Hyalomma* was the most prevalent, followed by genus *Rhipicephalus* and *Haemaphysalis*. Herein, unlike [2,3,8], *Hy. dromedarii* was the most prevalent in the region owing to the screening of a larger number of camels compared to other hosts. According to the studies performed in the region [2,3,8], *R. microplus* and *Ha. bispinosa* were the most prevalent tick species in the genus *Rhipicephalus* and genus *Haemaphysalis*, respectively.

The highest prevalence of *A. marginale* occurs in those regions where *R. microplus* is endemic [45]. This implies that *R. microplus* is one of the most competent vectors for *A. marginale*. Comparatively, *A. marginale* was highly detected in *Hy. dromedarii,* followed by *R. microplus* in the present study. This pathogen was also detected in four other tick species, including *Hy. anatolicum*, *Hy. dromedarii*, *R. sanguineus* and *Hy. scupense*. To the best of our knowledge, the detection of *A. marginale* in *R. sanguineus* is exceptionally rare [46], and this pathogen has not been detected in *Hy. scupense*. However, experimentally it has been demonstrated that this pathogen can be successfully transmitted by *R. sanguineus* [47,48]. Therefore, such unexpected outcomes need to be further evaluated because the presence of a pathogen DNA in a tick species does not ensure it as a biological vector. Moreover, a global increase in moments of infected/carrier livestock and/or tick-infested livestock across international borders can further worsen the situation regarding this pathogen [49].

For the host range of ticks, the resemblance among hosts’ ecology might be more significant than evolutionary similarity [50]. A wide host range was recorded for *Hy. anatolicum* that could be attributed to its two or three host life cycle with the infestation on different ungulates [51]. A comparatively wide host range was also noted for one host tick species such as *R. microplus*. This might be due to common practices in the study area, such as placing different hosts in the same shelter, overcrowded herds, and combined grazing.

Research has been focused on understanding the evolutionary history and taxonomy of ticks and TBPs using standard genetic markers [36,52,53]. The mitochondrial gene *cox1* has been considered an appropriate genetic marker for understanding tick phylogenetic relationships, especially at the species level [52]. The *16S rRNA* gene has also been considered a reliable marker for tick identification [36,52] and is of prime importance in evaluating bacterial phylogeny and taxonomy [54,55]. When taking these into account, the *cox1* sequences were obtained for eight tick species (*Hy. dromedarii*, *Hy. anatolicum*, *Hy. scupense*, *Hy. isaaci*, *Ha. bispinosa*, *R. microplus*, *R. sanguineus,* and *R. haemaphysaloides*). For the remaining four tick species (*R. turanicus*, *Ha. cornupunctata*, *Ha. montgomeryi,* and *Ha. sulcata*), we were able to obtain only *16S rRNA* sequences. The *A. marginale* associated with these ticks was molecularly assessed by targeting the partial *16S rRNA* gene. Except for *Ha. cornupunctata*, all other *Haemaphysalis* species were clustered with related species reported from the Oriental and neighboring Palearctic zoogeographical regions (Figure 5 and Figure 6). In the *cox1*-based phylogenetic tree, the monophyletic clade containing *Ha. bispinosa* was basal to the remaining ixodid tick species. In tick *16S rRNA*-based tree, the monophyletic clade having *Ha. sulcata* was at a basal position to all other hard tick species. The clade that had *Ha. montgomeryi* appeared as sister to the clade possessing both *Ha. obesa* and *Ha. parva*. Before the genetic data, based on morphological resemblance, these species were placed in the same subgenus *Segalia* of *Haemaphysalis* [56]. Due to the lack of previous genetic data, *Ha. cornupunctata* was displayed individually as sister taxa to the clade, which constitutes *Ha. punctata*. The closeness between these species has already been well established from morphological similarities; accordingly, they were assigned the same subgenus *Aboimisalis* of *Haemaphysalis* [6,56]. *Hyalomma* species were clustered with related species from Oriental, neighboring Palearctic, and Afrotropic regions. In the phylogenetic tree inferred from tick *cox1*, the clade formed by *Hy. anatolicum* clustered as sisters to the clade of *Hy. excavatum*. This concurs with the morphological resemblance among *Hy. anatolicum* and *Hy. excavatum*. The clade containing *Hy. dromedarii* was sister to the clade formed by *Hy. scupense* (jointly with seven other *Hyalomma* species). In the same phylogenetic tree, the clades of *Hy. scupense* and *Hy. asiaticum* were revealed as sister taxa. These studies were concordant with previous demonstrations [57]. *Hyalomma isaaci* clade appeared as a distinct species that did not support this species as a sub-species of *Hy. marginatum* [58,59,60] but supported this species as a valid species [31]. *Rhipicephalus* species clustered with the same species from Oriental and neighboring Palearctic regions. In the tick *cox1*-based phylogenetic tree, the *R. sanguineus* clade appeared to be sister to the *R. turanicus* clade, and both were jointly sister to the *R. rossicus* and *R. pumilio* clade. In the phylogenetic tree inferred from tick *16S rRNA*, *R. turanicus,* along with *R. sanguineus* and *R. linnaei,* appeared to be sister to *R. camicasi*. These mentioned *Rhipicephalus* species are included in the *R. sanguineus* species complex, and *R. sanguineus* from the temperate lineage was found closest to *R. turanicus* [11,61]. Following a previous study [62], different genetic groups were depicted within *R. haemaphysaloides* in the present phylogenetic analysis. *Rhipicephalus microplus* of clade-c was found close to *R. annulatus* [3,11,63]. In the phylogenetic tree based on bacterial *16S rRNA*, the clade of *A. marginale* and *A. ovis* clustered as a sister clade to the clade of *A. centrale*. This relatedness is also represented by their ecological and epidemiological aspects because these three species commonly infect ruminants [24].

## 5. Conclusions

In this study, 12 hard tick species were morphologically and molecularly identified; among them, four species (*Ha. bispinosa*, *Ha. cornupunctata*, *Hy. dromedarii*, and *Hy. isaaci*) were molecularly characterized for the first time from Pakistan. Notably, this is the first report providing *Ha. cornupunctata* genetic data and preliminary phylogenetic analysis. Furthermore, *A. marginale* was molecularly assessed in six tick species; among them, this pathogen was molecularly detected for the first time in four tick species (*Hy. dromedarii*, *Hy. scupense*, *R. sanguineus*, and *R. haemaphysaloides*) from Pakistan. Further studies should assess the genetic diversity of ticks and associated *Anaplasma* spp. in the country. This study might help in recognizing knowledge gaps and provide future direction to veterinary and health authorities in controlling ticks and *A. marginale*.

## Figures and Tables

**Table 1:** Primers used for the detection of ticks and associated *Anaplasma* spp.

Organism/Gene	Sequence (5′-3′)	Amplicon Size	PCR Condition	Ref.
**Ticks/*cox1***	*cox1* F, GGAACAATATATTTAATTTTTGG *cox1* R, ATCTATCCCTACTGTAAATATATG	801 bp	95 °C 5 min, 40× (95 °C 30 s, 55 °C 60 s, 72 °C 1 min), 72 °C 5 min	[35]
**Ticks/*16S rRNA***	16S+1, CCGGTCTGAACTCAGATCAAGT 16S−1, GCTCAATGATTTTTTAAATTGCTG	460 bp	95 °C 3 min, 40× (95 °C 30 s, 55 °C 60 s, 72 °C 1 min), 72 °C 7 min	[36]
***Anaplasma* spp./*16S rRNA***	Ehr-F2, AGAGTTTGATCCTGGCTCAG Ehr-R, AGTTTGCCGGGACTTYTTCT	1100 bp	95 °C 3 min, 35× (95 °C 30 s, 50 °C 30 s, 72 °C 1 min), 72 °C 7 min	[37]

**Table 2:** Prevalence of ticks and the detection rate of *Anaplasma marginale*.

Tick Species	Tick Life Stages			Total Ticks (%)	Ticks Subjected to PCR	Infested Hosts	*Anaplasma marginale*	
	Female • (%)	Male (%)	Nymph (%)				Positive Ticks	Infection Rate %
***Hy. dromedarii***	187 (47.9)	170 (43.6)	33 (8.5)	390 (20.8)	45	Camels, Sheep, Cattle	14	31.1
***Hy. anatolicum***	140 (47.6)	128 (43.5)	26 (8.9)	294 (15.7)	42	Cattle, Sheep, Goats, Dogs, Asian water buffaloes, Horses, Camels	10	23.8
***Hy. scupense***	103 (49.7)	86 (41.6)	18 (8.7)	207 (11.0)	33	Cattle, Asian water buffaloes, Horses	9	27.3
***Hy. isaaci***	33 (56.9)	19 (32.8)	6 (10.3)	58 (3.1)	15	Sheep, Cattle, Goats	0	0
***Ha. cornupunctata***	51 (47.7)	42 (39.3)	14 (13)	107 (5.7)	8	Sheep, Goats	0	0
***Ha. montgomeryi***	42 (48.3)	36 (41.4)	9 (10.3)	87 (4.6)	6	Goats, Sheep	0	0
***Ha. bispinosa***	26 (48.2)	18 (33.3)	10 (18.5)	54 (2.9)	8	Goats, Sheep	0	0
***Ha. sulcata***	21 (44.7)	17 (36.2)	9 (19.1)	47 (2.5)	7	Sheep, Goats	0	0
***R. microplus***	126 (48.1)	78 (29.8)	58 (22.1)	262 (14)	40	Cattle, Asian water buffaloes, Sheep, Goats, Dogs	12	30
***R. turanicus***	61 (50.4)	51 (42.2)	9 (7.4)	121 (6.5)	6	Sheep, Goats, Dogs, Horses	0	0
***R. sanguineus***	71 (52.2)	52 (38.2)	13 (9.6)	136 (7.3)	30	Dogs, Sheep, Goats	6	20
***R. haemaphysaloides***	53 (48.2)	45 (40.9)	12 (10.9)	110 (5.9)	30	Dogs, Sheep, Goats	6	20
**Total**	914 (48.8)	742 (39.6)	217 (11.6)	1873	314		54	17.2

**•** Count for fully, partially and unengorged.

## Data Availability

All the relevant data are within the manuscript.
